# UCell: Robust and scalable single-cell gene signature scoring

**DOI:** 10.1016/j.csbj.2021.06.043

**Published:** 2021-06-30

**Authors:** Massimo Andreatta, Santiago J. Carmona

**Affiliations:** a Ludwig Institute for Cancer Research, Lausanne Branch, and Department of Oncology, CHUV and University of Lausanne, Epalinges 1066, Switzerland; b Swiss Institute of Bioinformatics, Lausanne, Switzerland

**Keywords:** Single-cell, Gene signature, Module scoring, Cell type, Gene set enrichment

## Abstract

UCell is an R package for evaluating gene signatures in single-cell datasets. UCell signature scores, based on the Mann-Whitney U statistic, are robust to dataset size and heterogeneity, and their calculation demands less computing time and memory than other available methods, enabling the processing of large datasets in a few minutes even on machines with limited computing power. UCell can be applied to any single-cell data matrix, and includes functions to directly interact with Seurat objects. The UCell package and documentation are available on GitHub at https://github.com/carmonalab/UCell.

## 1 Introduction

In single-cell RNA-seq analysis, gene signature (or “module”) scoring constitutes a simple yet powerful approach to evaluate the strength of biological signals, typically associated to a specific cell type or biological process, in a transcriptome. Thousands of gene sets have been derived by measuring transcriptional differences between different biological states or cell phenotypes, and are collected in public databases such as MSigDB [1]. More recently, large-scale efforts to construct single-cell atlases [2], [3] are providing specific gene sets that can be useful to discriminate between cell types. For example, Han et al. have used single-cell RNA sequencing to quantify cell type heterogeneity in different tissues and to define gene signatures for >100 human and murine cell types [3]. Given such a gene set, signature scoring aims at quantifying the activity of the genes in the set, with the goal to characterize cell types, states, active biological processes or responses to environmental cues. The Seurat R package [4] is one of the most comprehensive and widely used frameworks for scRNA-seq data analysis. Seurat provides a computationally efficient gene signature scoring function, named AddModuleScore, originally proposed by Tirosh et al. [5]. However, because genes are binned based on their average expression across the whole dataset for normalization purposes, the method generates inconsistent results for the same cell depending on the composition of the dataset. Inspired by the AUCell algorithm implemented in SCENIC [6], we propose UCell, a gene signature scoring method based on the Mann-Whitney U statistic. UCell scores depend only on the relative gene expression in individual cells and are therefore not affected by dataset composition. We provide a time- and memory-efficient implementation of the algorithm that can be seamlessly incorporated into Seurat workflows.

## 2 Methods

UCell calculates gene signature scores for scRNA-seq data based on the Mann-Whitney U statistic [7]. Given a *g × c* matrix ***X*** of numerical values (e.g. gene expression measurements) for *g* genes in *c* cells, we first calculate the matrix of relative ranks ***R*** by sorting each column in ***X***; in other words, we calculate a ranked list of genes for each cell in the dataset. Because in scRNA-seq not all molecules in the original sample are observed, transcript counts matrices contain many zeros, resulting in a long tail of bottom-ranking genes. To mitigate this uninformative tail, we set *r_g,c_* = *r_max_* + 1 for all *r_g,c_* > *r_max_*, with *r_max_* = 1500 by default (matching typical thresholds used for quality control for minimum number of genes detected). To evaluate a gene signature ***s*** composed of *n* genes (*s_1_ ,*…*,s_n_*), we calculate a UCell score U’ for each cell *j* in ***X*** with the formula:

$$U_{j}^{\text{'}}=1-\frac{U_{j}}{n∙r_{\text{max}}}$$

where U is the Mann-Whitney U statistic calculated by:

$$U_{j}=\sum_{i=1}^{n}r_{i,j}^{\text{'}}-\frac{n(n+1)}{2}$$

and ***R′*** is obtained by sub-setting ***R*** on the genes in signature ***s***.

We note that the U statistic is closely related to the area-under-the-curve (AUC) statistic for ROC curves [8], therefore we expect UCell scores to correlate with methods based on AUC scores such as AUCell [6]. Internally, UCell uses the *frank* function from the *data.table* package [9] for efficient ranks computations. Large datasets are automatically split into batches of reduced size, which can be processed serially (minimizing memory usage) or in parallel through the *future* package [10] (minimizing execution time) depending on the available computational resources.

## 3 Results

**Fig. 1:**
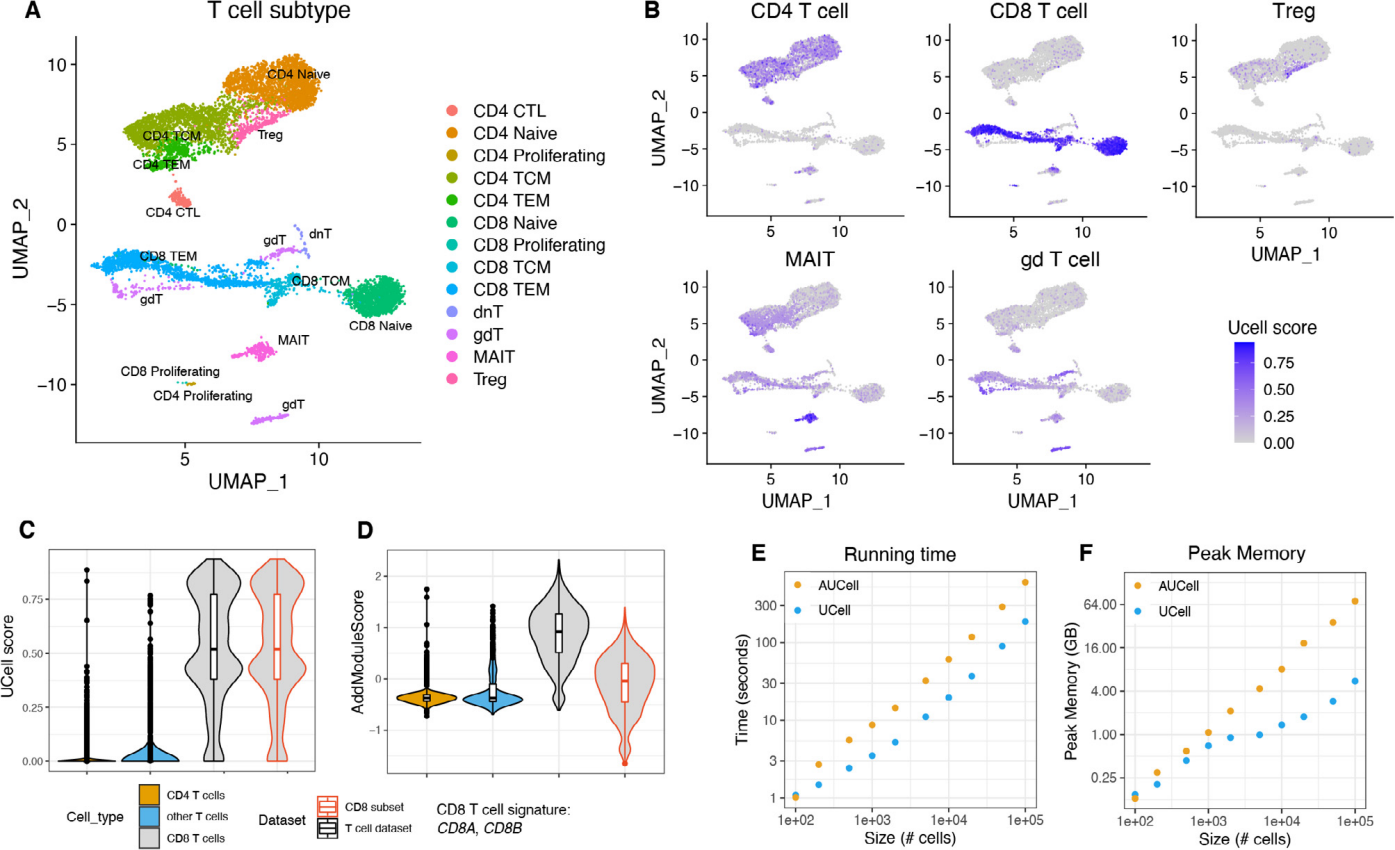
Evaluating T cell signatures using UCell. A) UMAP representation of T subsets from the single-cell dataset by Hao et al. [11]. B) UCell score distribution in UMAP space for five gene signatures (listed in Table 1) evaluated using UCell. C-D) Comparison of UCell score (C) and Seurat’s AddModuleScore (D) distributions for a two-gene CD8 T cell signature (*CD8A*, *CD8B*), evaluated on the complete T cell dataset (black outlines), or on the subset of CD8 T cells only (red outlines); UCell scores for CD8 T cell have the same distribution in the complete or subset dataset, while AddModuleScores are highly dependent on dataset composition. E-F) Running time (E) and peak memory (F) for UCell and AUCell (which produces similar results) on datasets of different sizes show that UCell is about three times faster and requires up to ten times less memory on large (>10^4^) single-cell datasets. (For interpretation of the references to colour in this figure legend, the reader is referred to the web version of this article.)

UCell is an R package for the evaluation of gene signature enrichment designed for scRNA-seq data. Given a gene expression matrix or Seurat object, and a list of gene sets, UCell calculates signature scores for each cell, for each gene set. In the following illustrative example, we applied UCell to a single-cell multimodal dataset of human blood T cells [11], which were annotated by the authors using both gene (scRNA-seq) and cell surface marker expression (CITE-seq) (Fig. 1A). Provided a set of T cell subtype-specific genes (Table 1), UCell helps interpreting clusters in terms of signature enrichment in low-dimensional spaces such as the UMAP (Fig. 1B). Importantly, UCell scores are based on the relative ranking of genes for individual cells, therefore they are robust to dataset composition. Evaluating a CD8 T cell signature on the full dataset or on CD8 T cells only, results in identical score distributions for CD8 T cells in the two settings (Fig. 1C). Conversely, AddModuleScore from Seurat normalizes its scores against the average expression of a control set of genes across the whole dataset, and is therefore dependent on dataset composition. CD8 T cells analyzed in isolation or in the context of the full T cell dataset are assigned highly different AddModuleScore scores, with median ~1 in the full dataset and median ~0 for the CD8 T cell subset (Fig. 1D). Another widely-used method for single-cell signature scoring, AUCell [6], is also based on relative rankings and therefore has the same desirable property as UCell of reporting consistent scores regardless of dataset composition. Compared to AUCell, UCell is about three times faster (Fig. 1E) and uses significantly less memory (Fig. 1F). For example, AUCell requires over 64 GB of RAM to process 100,000 single-cells, while UCell uses only 5.5 GB of peak memory (Fig. 1F), making it suitable even for machines with limited computing power.

**Table 1:** Gene signatures for T cell subsets in Fig. 1.

T cell type	Gene set
CD4 T cell	*CD4*, *CD40LG*
CD8 T cell	*CD8A*, *CD8B*
Treg	*FOXP3*, *IL2RA*
MAIT	*KLRB1*, *SLC4A10*, *NCR3*
gd T cell	*TRDC*, *TRGC1*, *TRGC2*, *TRDV1*

UCell is available as an R package at https://github.com/carmonalab/UCell, and is accompanied by vignettes for signature scoring and for seamless integration with Seurat pipelines. Source code to reproduce the results in this manuscript is available at the following repository: https://gitlab.unil.ch/carmona/UCell_demo.

## Funding

This research was supported by the 10.13039/501100001711Swiss National Science Foundation (SNF) Ambizione grant 180010 to SJC.

## CRediT authorship contribution statement

**Massimo Andreatta:** Conceptualization, Methodology, Software, Formal analysis, Visualization, Writing - original draft, Writing - review & editing. **Santiago J. Carmona:** Conceptualization, Methodology, Software, Formal analysis, Writing - original draft, Writing - review & editing, Funding acquisition.

## Declaration of Competing Interest

The authors declare that they have no known competing financial interests or personal relationships that could have appeared to influence the work reported in this paper.
